# Rupture of Non-aneurysmal Mycotic Thoracic Aortic Arteritis Caused by Campylobacter fetus

**DOI:** 10.7759/cureus.50244

**Published:** 2023-12-09

**Authors:** Shinya Ikeda, Yuji Nishimoto, Masanao Toma, Yukihito Sato

**Affiliations:** 1 Department of Pharmacology, Shiga University of Medical Science, Otsu, JPN; 2 Department of Cardiology, Hyogo Prefectural Amagasaki General Medical Center, Amagasaki, JPN

**Keywords:** mycotic abdominal aortic aneurysm, mycotic thoracic aortic arteritis, non-aneurysmal mycotic aortic arteritis, rupture, campylobacter fetus

## Abstract

Campylobacter fetus (C. fetus) demonstrates a preference for vascular tissue and is an infrequent etiology of mycotic aortic arteritis (MAA), mostly occurring in the abdominal aorta. MAA characteristically has a rapid progression to aneurysm formation and subsequently, to aortic rupture. We present a 73-year-old woman with non-aneurysmal mycotic thoracic aortic arteritis (MTAA) complicated with a rupture caused by C. fetus. She presented after four days of pain in the lower abdomen. Contrast-enhanced computed tomography revealed non-aneurysmal descending thoracic aorta arteritis and an abdominal aorta aneurysm, and the blood cultures were positive for C. fetus. Antibiotic therapy relieved the abdominal pain. However, eight days after the antibiotic therapy, she died because of a rupture of the non-aneurysmal MTAA. The non-aneurysmal MTAA caused by C. fetus ruptured while the infection was being treated with appropriate antibiotics, and there was no sign of arterial dilatation. An early open or endovascular repair after a short pre-operative antibiotic therapy may be required for non-aneurysmal MAA caused by C. fetus. More cases of non-aneurysmal MAA caused by C. fetus are needed to determine the clinical course and to decide the treatment strategy.

## Introduction

Campylobacter fetus (C. fetus) demonstrates a preference for vascular tissue and is a rare but well-known cause of mycotic aortic arteritis (MAA). Most of the previous cases of MAA caused by C. fetus occurred in the abdominal aorta [1]. MAA progresses to an aortic aneurysm and subsequently to an aortic rupture. However, we present a patient who developed non-aneurysmal MAA in the descending thoracic aorta caused by C. fetus that ruptured without dilation of the aorta.

## Case presentation

A 73-year-old woman with hypertension presented with a four-day history of lower abdominal pain and a two-day history of chills. The worsening abdominal pain caused her to visit our emergency department. She had smoked 30 cigarettes per day and had no history of any immunodeficiency. She did not have any contact with animals or a history of eating raw meat.

**Figure 1:**
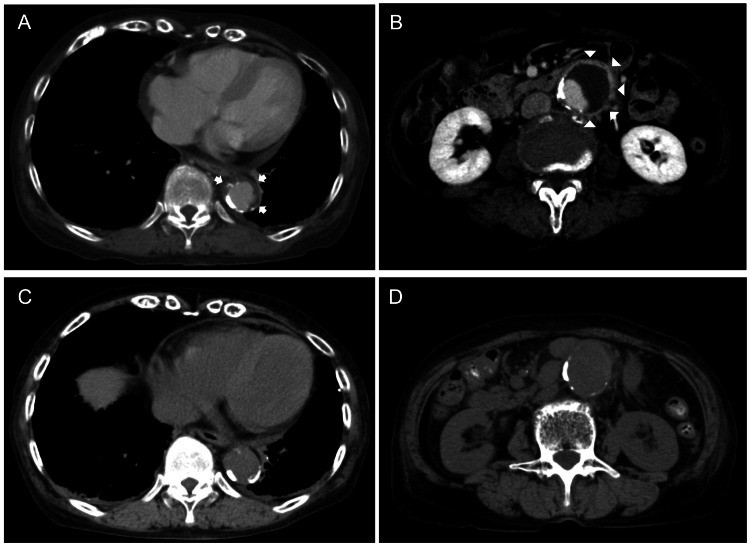
Axial views of the CT angiogram of the thorax and abdomen on admission and plain CT on the third hospital day. A) Descending thoracic aortic arteritis on admission. The arrows show an ill-defined soft tissue abnormality measuring 30 mm × 27 mm. B) Infrarenal abdominal aortic aneurysm on admission. The arrowheads show an ill-defined soft tissue abnormality measuring about 42 mm × 41 mm. C) Descending thoracic aorta on the third hospital day measuring 29 mm × 27 mm. D) Infrarenal abdominal aortic aneurysm on the third hospital day measuring 42 mm × 39 mm. CT, computed tomography.

On admission, she had a temperature of 36.4°C and normal blood pressure and heart rate. She had an elevated white cell count at 15.7 x 103 cells/cm^3^ and a high C-reactive protein (CRP) level at 20.09 mg/dL but a normal procalcitonin level at 0.22 ng/mL. Contrast-enhanced computed tomography (CT) revealed an infrarenal aortic aneurysm of 41 mm with periarteritis and descending aorta arteritis of 27 mm with a subtle enhancement of the vasa vasorum (Figures 1A, 1B). Based on these results, the patient was diagnosed with inflammatory arteritis in the descending thoracic and abdominal aorta. However, on the third hospital day, her blood cultures were positive for Campylobacter. She was diagnosed with MAA in the descending thoracic and abdominal aorta due to Campylobacter. Antibiotic therapy with meropenem 3 g/day followed by a surgical repair was selected because the diameter of the aneurysm had not changed on repeat CT obtained on the third hospital day (thoracic aorta: 27 mm, abdominal aorta: 39 mm) (Figures 1C, 1D), an open repair for multiple infectious sites was deemed to carry a higher risk of mortality and morbidity, and an endovascular repair during a bloodstream infection could worsen the infection. The antibiotic therapy with meropenem relieved her symptoms. However, because the CRP level decreased, but not sufficiently, the timing of a semi-urgent surgical repair was discussed.

**Figure 2:**
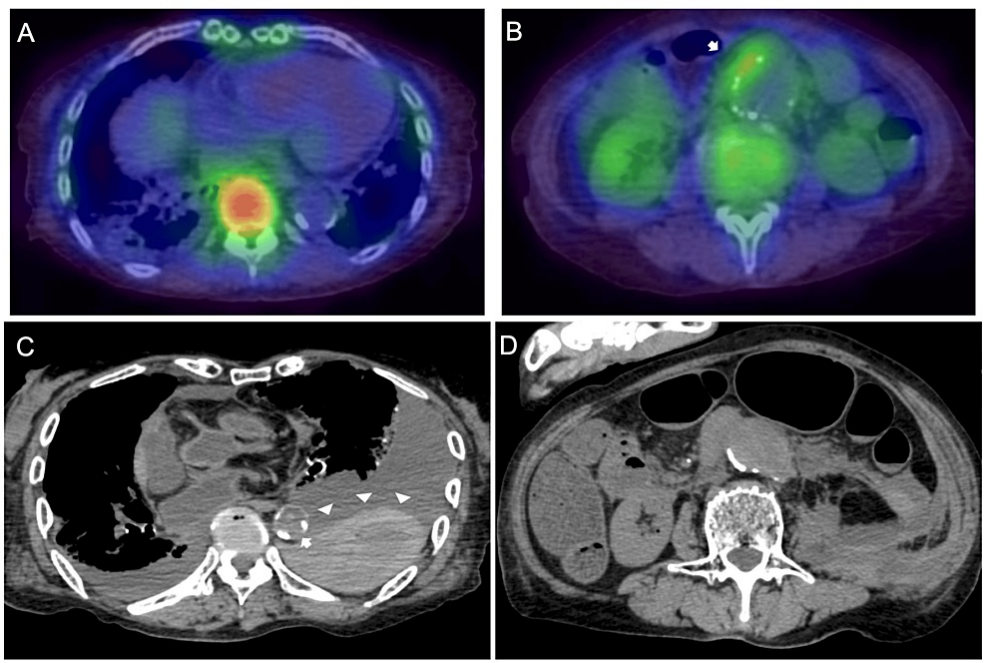
Axial views of gallium scintigraphy of the thorax and abdomen on the ninth hospital day and autopsy CT imaging of the thorax and abdomen. A) Descending thoracic aorta on gallium scintigraphy. No inflammation was detected in the thoracic aorta. B) Infrarenal abdominal aortic aneurysm on gallium scintigraphy. The arrow shows inflammation in the abdominal aortic aneurysm. C) Descending thoracic aorta from the autopsy imaging. The arrow shows that the arterial wall was disrupted, and the arrowheads show a lot of thoracic bleeding. D) Infrarenal abdominal aortic aneurysm from the autopsy imaging. CT, computed tomography.

**Figure 3:**
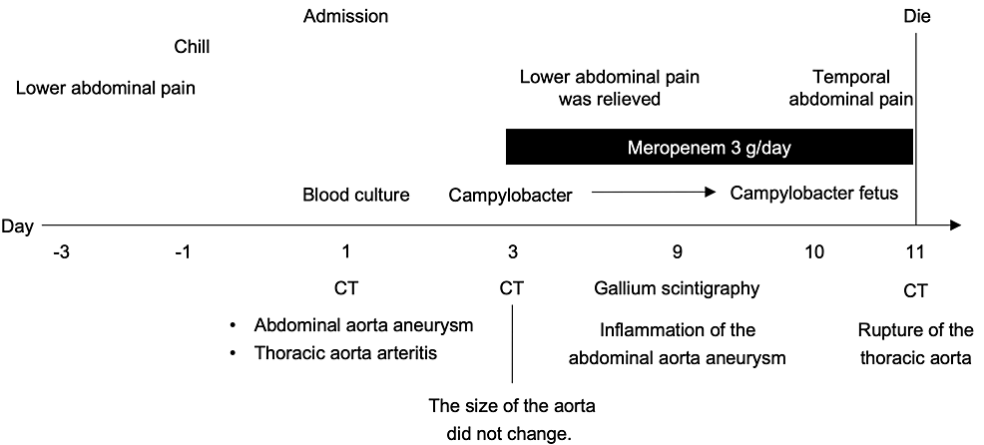
Timeline. Relevant data from this episode of the care given organized as a timeline. CT, computed tomography.

Gallium scintigraphy on the ninth hospital day revealed no apparent further dilatation of the thoracic and abdominal aorta, inflammation in the abdominal aorta, and no inflammation of the thoracic aorta (Figures 2A, 2B). We put off the semi-urgent surgical repair and examined the diameter of the thoracic and abdominal aorta with CT a few days later. On the 10th hospital day, she had a temperature of 36.7°C, a blood pressure of 127/79 mmHg, and a heart rate of 80 beats per minute. Blood cultures obtained at the time of admission identified that the bacterium was C. fetus. On the 11th hospital day, she suffered from temporal abdominal pain with a blood pressure of 162/90 mmHg. After 15 minutes, she suddenly died. Autopsy CT imaging demonstrated an intrathoracic bleed with rupture of the mycotic thoracic aortic arteritis (MTAA), but no rupture of the mycotic abdominal aortic aneurysm (Figures 2C, 2D). The timeline is presented in Figure 3.

## Discussion

The main findings of the current case were as follows: 1) MAA with C. fetus occurred in the descending thoracic aorta. 2) The non-aneurysmal MAA caused by C. fetus ruptured under appropriate antibiotic therapy with no dilatation of the aorta.

MAA is caused by several types of bacteria [2]. The most cultured bacteria include Salmonella (33%), Staphylococcus (16%), and Streptococcus (10%). Campylobacter species account for 0.7% of mycotic aneurysms. Most of the previous cases of MAA caused by C. fetus occurred in the abdominal aorta [1]. In addition, MAA caused by C. fetus in an internal iliac [3], femoral [4], and popliteal [5] artery has also been reported. However, to our knowledge, there have been no reports of MAA with C. fetus in the descending thoracic aorta. The mortality rate of MTAA is higher than MAA in the abdominal aorta [2]. We should collect more cases of MTAA caused by C. fetus in order to determine the accurate clinical course of MTAA caused by C. fetus, including therapeutic strategies.

The non-aneurysmal MTAA caused by C. fetus ruptured under appropriate antibiotic therapy without dilatation of the aorta. The overall mortality of C. fetus MAA is 20-30% [6], but the mortality approaches 100% if a rupture occurs before surgical intervention can be conducted [7]. No consensus has yet been reached on the optimal duration of pre-operative antibiotics [8] and the timing of surgery. Generally, a two- to six-week pre-operative antibiotic therapeutic interval has been suggested unless the patient's condition mandates emergency surgery. Emergency surgery has been performed in patients with uncontrolled infections (persistent fever or septic shock) or evidence of an impending aortic rupture (persistent pain, shock, or an enlarged pseudoaneurysm formation on repeated imaging studies) [2,9]. In this case, we estimated the risk of a rupture was low because the size and shape of the aorta had not changed over time and the antibiotic therapy relieved the symptoms in the patient. The low risk of a rupture led us to the standard pre-operative antibiotic therapy until the blood cultures were negative and the inflammation in the aorta disappeared. However, non-aneurysmal MAAs caused by several types of bacteria, such as Salmonella and Staphylococcus, can rupture despite the size of the aneurysm [10]. Moreover, Kan et al. reported that pre-operative antibiotic treatment for more than three days reduced aneurysm-related mortality (Odds ratio: 0.2, 95% CI: 0.04-0.96, p = 0.053) [11]. This clinical course showed that an early open or endovascular repair with short pre-operative and life-long post-operative antibiotic therapy may be required for non-aneurysmal MAA caused by C. fetus like other bacteria, such as Salmonella and Staphylococcus [10]. We should collect more cases of non-aneurysmal MAA caused by C. fetus to clarify the efficiency of early surgical intervention in the low rupture-risk group.

In this case, we have one reflection point. The point was the initial antibiotic therapy. On admission, the patient was diagnosed with inflammatory arteritis and was not treated for two days because her procalcitonin levels were low because a previous review paper showed that the level of procalcitonin < 0.25 does not recommend antibiotics [12]. However, she should have initially been treated with a broad-spectrum antibiotic. Nevertheless, given the high mortality rate of MAA, it was likely that the broad-spectrum antibiotic therapy given from admission could not have saved her life without early surgical repair [6,7].

## Conclusions

To our knowledge, this was the first case of MAA in the descending thoracic aorta caused by C. fetus. The non-aneurysmal MTAA caused by C. fetus ruptured within the standard duration of pre-operative antibiotic therapy. An early surgical repair with a short pre-operative antibiotic therapy may be beneficial for non-aneurysmal MAA caused by C. fetus even under no sign of a rupture. We should collect more cases of non-aneurysmal MAA caused by C. fetus.
